# Citius, Altius, Fortius: Performance in a Bottle for CAR T-Cells

**DOI:** 10.33696/haematology.1.015

**Published:** 2020

**Authors:** Asma Ayari, Roddy S. O’Connor

**Affiliations:** 1 Nucleus Biologics, LLC, San Diego, CA, USA; 2 Center for Cellular Immunotherapies, Perelman School of Medicine at the University of Pennsylvania, Philadelphia, PA, USA; 3 Department of Pathology and Laboratory Medicine, Perelman School of Medicine of the University of Pennsylvania, Philadelphia, PA, USA

The renewed interest in understanding how activated T cells alter their metabolism to support their growth and differentiation has led to several innovative advances in synthetic biology; culminating in a number of genetic and pharmacologic approaches aimed at improving the antitumor function of adoptively transferred T cells. Indeed, the growing field of immunometabolism has accelerated rapidly giving rise to exciting discoveries and exploratory studies revealing how T cells balance metabolic adaptations in response to intrinsic and extrinsic regulatory cues. Central to this body of work, we showed how chimeric antigen receptors (CAR)-induced metabolic reprogramming is an important determinant of efficacy and clinical outcome in blood-based malignancies [1]. CAR T-cell production involves a rigorous, and systematic *ex-vivo* expansion regime involving activation; genetic modification with either a CAR or tumor-specific T cell receptor (TCR); and proliferative phase which often lasts 14 days. As CAR T-cells progressively differentiate over time in culture, a process that impairs engraftment and potency following adoptive transfer, it’s surprising that the metabolic composition of clinical grade cell culture mediums has been largely understudied. Increasing evidence suggests that subtle adjustments in medium formulation can have a dramatic impact on T cell bioactivity and anti-tumor function in several preclinical models of cancer. In a recent article, we provide direct evidence that standard medium formulations are suboptimal, and introduce a serum-free, concentrated, platelet extract as a superior alternative to human serum in clinical-grade medium for CAR T-cells [2].

What prompted our study? With the increased number of CAR-T cell clinical trials worldwide, a critical inflection point will be reached where the demand for human serum exceeds its supply. A disruption in the supply chain, and a decline in human serum availability will provide an insurmountable barrier to CAR T-cell immunotherapies against cancer. In the course of our studies, we identified a serum-free, concentrated, platelet extract, “Phx”, that optimized gene transfer into primary human T cells, increases effector function *in vitro,* and improves the antitumor potency of CAR-T cells *in vivo*. Future efforts will uncover if Phx can substitute for HS in freezing medias. A model describing our findings is presented in Figure 1.

Using an LC-MS-based screen, we show that carnosine is enriched in Phx relative to human serum. Carnosine (beta-alanyl-L-histidine) is a dipeptide molecule, highly concentrated in skeletal muscle and brain. Carnosine has been shown to buffer proton accumulation, neutralize free radicals, and chelate metal ions [3]. We provide novel evidence that carnosine enhances the metabolic fitness of activated T cells. It’s well established that activated T cells undergo a metabolic shift to glycolysis support their increased energetic and biosynthetic demands. Committing to glycolysis centers on a paradoxical axis: metabolizing glucose to lactic acid supports high rates of ATP replenishment but creates an acidic milieu that impairs overall functional competence. Carnosine has an intrinsic ability to neutralize the increase in extracellular acidification, skewing metabolism towards an oxidative phenotype. Competition for oxygen is an important determinant of efficacy in adoptive immunotherapies. Delgoffe and coworkers showed how CD8^+^ TiL cells were particularly vulnerable to oxygen depletion in xenograft models of melanoma [4]. Compared to T cells that evacuated solid tumors and migrated to nearby lymph nodes, the ability of CD8^+^ TILs to consume oxygen is suppressed and mitochondrial function is impaired. Restoring their oxidative state enhances the efficacy of anti-PD-1 immune checkpoint blockade [5] Our findings suggest that carnosine confers a superior oxidative state to cultured T cells; an effect that often underlies improved mitochondrial function. Our study implicates a novel role for carnosine in medium formulations designed for adoptive immunotherapies. Phx also contains higher levels of monosaccharide derivatives including glucosamine-6-phosphate, D-sedoheptulose-1/7 biphosphate, and glucose-1 phosphate, as well as phospholipid and nucleotide derivatives compared to human serum. How their abundance impacts T cells during the *ex-vivo* expansion phase is unknown.

Bypassing the local restrictions of an acidic milieu using carnosine gives rise to T cells with superior metabolic fitness. Circulating T cells are exposed to Carnosinase-1 (CN1) which is abundant in serum and cleaves the dipeptide by hydrolysis. In its free form, L-histidine has weak buffering capacity at physiologic ranges. For this reason, supplying carnosine in its free form, or in Phx, to T cells during ex-vivo culture is an ideal strategy to improve their performance following adoptive transfer. As tumor cells establish acidic environments to suppress T cell cytolytic activity [6], the findings from our work suggest that Carnosine “loaded” T cells may have a performance edge in these environments.

There are inherent challenges in delivering CAR transgenes to healthy volunteer as well as patient T cells. Polybrene and Chloroquine which bridge the virus to the T cell membrane, and restrict virus degradation in the lysosome, respectively, are commonly used to enhance retroviral-mediated gene delivery in stem cells. These are not widely used in T cell therapies. Here, Phx significantly enhanced lentiviral-mediated gene expression. These findings have important implications for reducing lentiviral production costs in CAR –T manufacturing process. Interestingly, carnosine was an important factor in Phx supporting lentiviral gene transfer into activated T cells. Lentiviral infection efficiencies increased from 31% to 43% when standard medium was supplemented with 30 mM carnosine. Future studies will determine the potential benefit of raising carnosine levels further in Phx-conditioned medium.

The components of medium used to fuel the expansion and differentiation of T cells for adoptive immunotherapies should be critically evaluated. Subtle changes in media formulation such as changing protein source from human serum to Phx has a dramatic impact on the effectiveness of T cell therapies. Using a human xenograft model of neuroblastoma, the anti-tumor function of GD-2-specific CAR T-cells was significantly enhanced in Phx relative to HS. Examining circulating lymphocyte numbers revealed that the overall persistence of adoptively transferred T cells was also significantly higher in Phx vs HS. These findings add to the growing body of work showing how metabolic reprogramming events during the *ex– vivo* expansion phase have immediate translational relevance for the clinical sector. OT- 1 T cells expanded in medium conditioned with L-arginine had increased anti-tumor function in a B16 murine model of melanoma [7]. Loss-of-function approaches show that pharmacologic inhibition of glycolysis enhances the differentiation state, persistence, and anti-tumor function, of adoptively transferred T cells [8,9]. Limiting activity of the glycolytic enzyme LDHA during T cell expansion led to increased efficacy in murine models of cancer [10]. Pharmacologic inhibiting P38 MAPK improved the efficacy of mouse anti-tumor T cells [11]. Implicit in these findings is that glycolysis supports effector function, and limiting effector differentiation prior to adoptive transfer yields progeny with superior engraftment following infusion. Complementing these results further, a recent paper showed how transient glucose restriction led to superior anti-tumor function following adoptive T cell transfer [12].

Adoptive cell therapies rely on the ability of activated T cells to undergo robust proliferation and differentiate into effector cell with cytolytic activity against cancer. Heterogeneity within the differentiating population ensure that a subset of T cells develop into memory cells, a stemlike lineage necessary for long-lasting immunity. Memory T cells are metabolically distinct from effector cells and can be distinguished by an increased mitochondrial content [1,13], and energy generating capacity [1,14]; while the study itself doesn’t identify a clear impact of Phx on the composition of naïve-like, central memory, or effector memory subsets (at least using standard flow cytometry panels), further analyses with RNA-Seq may reveal unique differentiation patterns underlying the superior performance of these CAR T-cells.

Optimizing media for cellular therapies has appeal across a broad clinical sector. Reconfiguring medium formulations to enhance T cell persistence, cytolytic function, and tumor clearance is an important priority in the CAR T-cell domain. As seen in this article, changing protein source from human serum to Phx, had a profound impact on cytolytic function in vitro and anti-tumor potency *in vivo*. By challenging the existing paradigm, and questioning the necessity of serum in current medium formulations, the article highlights an underappreciated role of medium formulation for enhancing cell therapies against cancer.

## Figures and Tables

**Figure 1:**
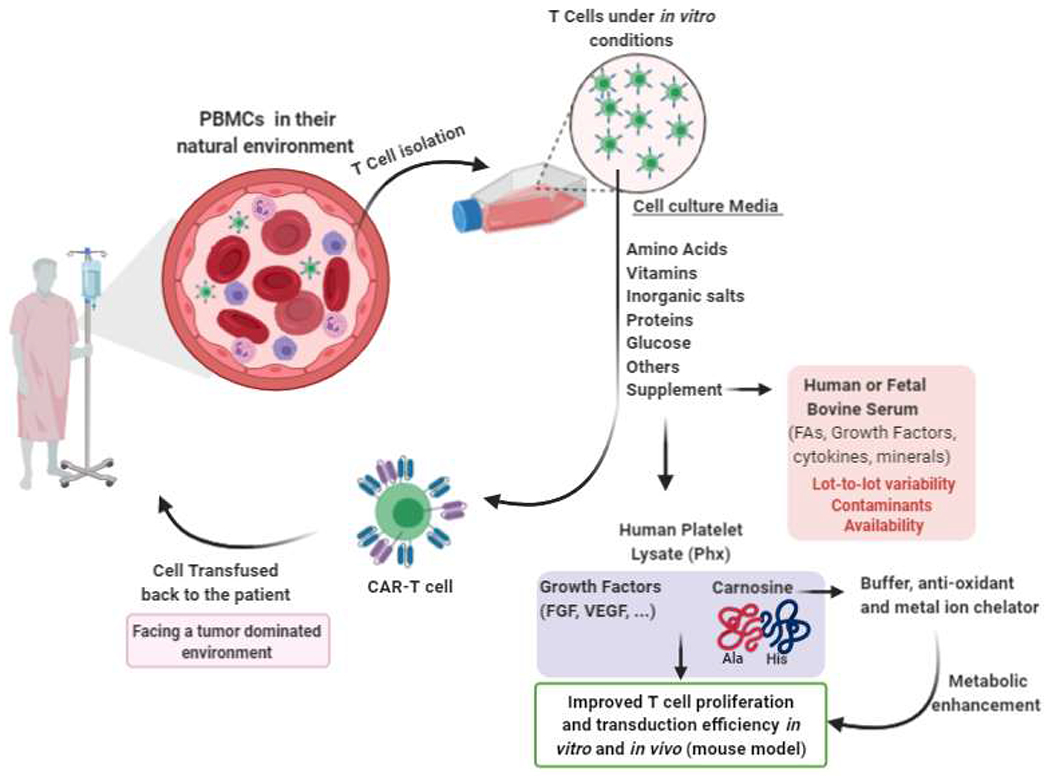
*In vitro* improvement of T cell proliferation and transduction efficiency in serum-free media supplemented with Phx (platelet lysate).
